# Breast carcinoma and Lynch syndrome: molecular analysis of tumors arising in mutation carriers, non-carriers, and sporadic cases

**DOI:** 10.1186/bcr3205

**Published:** 2012-06-12

**Authors:** Johanna E Lotsari, Annette Gylling, Wael M Abdel-Rahman, Taina T Nieminen, Kristiina Aittomäki, Marjukka Friman, Reino Pitkänen, Markku Aarnio, Heikki J Järvinen, Jukka-Pekka Mecklin, Teijo Kuopio, Päivi Peltomäki

**Affiliations:** 1 Department of Medical Genetics, Biomedicum Helsinki, P.O.Box 63 (Haartmaninkatu 8), University of Helsinki, Helsinki, Finland, FIN-00014; 2 College of Health Sciences, University of Sharjah, P.O. 27272 Sharjah, United Arab Emirates; 3 Department of Clinical Genetics, Helsinki University Central Hospital, P.O. Box 160 (Meilahdentie 2), Helsinki, Finland, FIN-00029; 4 Department of Pathology, Jyväskylä Central Hospital, (Keskussairaalantie 19), Jyväskylä, Finland, FIN-40620; 5 Department of Surgery, Jyväskylä Central Hospital, (Keskussairaalantie 19), Jyväskylä, Finland, FIN-40620; 6 Department of Surgery, Helsinki University Central Hospital, P.O Box 340 (Haartmaninkatu 4), Helsinki, Finland, FIN-00029

## Abstract

**Introduction:**

Breast carcinoma is the most common cancer in women, but its incidence is not increased in Lynch syndrome (LS) and studies on DNA mismatch repair deficiency (MMR) in LS-associated breast cancers have arrived at conflicting results. This study aimed to settle the question as to whether breast carcinoma belongs to the LS tumor spectrum.

**Methods:**

MMR status and epigenetic profiles were determined for all available breast carcinomas identified among 200 LS families from a nation-wide registry (23 tumors from mutation carriers and 18 from non-carriers). Sporadic breast carcinomas (n = 49) and other cancers (n = 105) from MMR gene mutation carriers were studied for comparison.

**Results:**

The proportion of breast carcinomas that were MMR-deficient based on absent MMR protein, presence of microsatellite instability, or both was significantly (*P *= 0.00016) higher among breast carcinomas from mutation carriers (13/20, 65%) compared to non-carriers (0/14, 0%). While the average age at breast carcinoma diagnosis was similar in carriers (56 years) and non-carriers (54 years), it was lower for MMR-deficient versus proficient tumors in mutation carriers (53 years versus 61 years, *P *= 0.027). Among mutation carriers, absent MMR protein was less frequent in breast carcinoma (65%) than in any of seven other tumor types studied (75% to 100%). Tumor suppressor promoter methylation patterns were organ-specific and similar between breast carcinomas from mutation carriers and non-carriers.

**Conclusions:**

Breast carcinoma from MMR gene mutation carriers resembles common breast carcinoma in many respects (for example, general clinicopathological and epigenetic profiles). MMR status makes a distinction: over half are MMR-deficient typical of LS spectrum tumors, while the remaining subset which is MMR-proficient may develop differently. The results are important for appropriate surveillance in mutation carriers and may be relevant for LS diagnosis in selected cases.

## Introduction

Germline mutation in one of four genes with DNA mismatch repair (MMR) function, *MLH1, MSH2, MSH6*, and *PMS2*, causes susceptibility to cancers of multiple organs known as Lynch syndrome (LS) [1]. Among all cancers, those of the colon and rectum, endometrium, small bowel, ureter and renal pelvis have the highest relative risk compared to the general population, which is why they are considered to be part of the LS tumor spectrum according to the Amsterdam criteria II [2]. Among extracolonic tumors accepted as LS spectrum tumors when the Amsterdam II criteria were formulated, cancer of the renal pelvis had the lowest relative risk (14) [3].

Breast cancer is the most common cancer among women worldwide [4]. It is presently not included in the LS tumor spectrum because its incidence has not been found to be elevated in LS patients, whether carriers of *MLH1 *or *MSH2 *mutation [5-8] or *MSH6 *mutation [9]. A few deviating reports exist. A study on Australian LS patients revealed a significantly increased standardized incidence ratio (14.77) for breast cancer in *MLH1*, but not *MSH2 *mutation carriers [10]. The standardized incidence ratio for breast cancer was also elevated (3.95) in a prospective investigation of a cohort of 446 unaffected carriers of *MLH1, MSH2, MSH6*, and *PMS2 *mutation over a median follow-up of five years [11]. Furthermore, among Brazilian families meeting the most stringent clinical (Amsterdam I) criteria for LS [12] but with no mutation data available, breast cancer was the most frequent extracolonic cancer in women, even exceeding the frequency of endometrial cancer [13].

As an additional approach to decide whether breast cancer is a LS spectrum tumor or not, breast cancers arising in LS patients have been studied for microsatellite instability (MSI) or MMR protein expression since deficient MMR is a hallmark of LS. As observed originally, the absence of MMR defects appeared to exclude breast cancer as an integral tumor of LS [14] whereas subsequent studies report the occurrence of MMR defects in breast carcinomas from MMR gene mutation carriers with frequencies ranging between 44% and 75% based on MSI and/or aberrant MMR protein expression [8,15-19]. Frequencies of MMR defects reported in the latter studies exceed those independently observed for sporadic breast carcinomas (0 to 20%) [20,21] suggesting that MMR deficiency is an important factor in breast cancer development in LS.

Given the conflicting results from previous studies, we collected all available LS-associated breast carcinomas through the nation-wide Hereditary Colorectal Cancer Registry of Finland. Molecular profiles in breast carcinomas from mutation carriers were compared to those from proven non-carriers (phenocopies) from mutation-positive families and to sporadic breast carcinomas from the same population; additionally, a large collection of other LS-associated tumors from mutation carriers was included in the analysis. Molecularly, breast carcinomas from LS mutation carriers were divided into two subgroups paralleled by some clinical differences (notably, different ages at onset) as will be described below.

## Materials and methods

### Patients and samples

The more than 200 LS families currently included in the nation-wide Hereditary Colorectal Cancer Registry of Finland were evaluated to identify cases with breast cancer. All available breast tumors including 41 tumors from 37 individuals who belonged to families with *MLH1, MSH2*, or *MSH6 *mutations were subsequently collected (Table 1). Twenty-three breast tumors originated from 19 mutation carriers and included 11, five, and seven tumors from carriers of *MLH1, MSH2*, and *MSH6 *mutation, respectively (please see Table 2 for mutation descriptions). Eighteen breast tumors were obtained from 18 non-carriers who belonged to families with *MLH1 *(15 tumors), *MSH2 *(two tumors), and *MSH6 *mutations (one tumor). Four families contributed at least one breast cancer from a carrier and one from a non-carrier. Nine breast tumors originated from carriers of a prevalent founder mutation (mutation 1) [22] and the same predisposing mutation underlay the families of ten non-carriers with breast cancer. Forty-nine sporadic breast tumors, selected from a larger consecutive series to represent the two main histological types, ductal (n = 30) and lobular (n = 19), and to match the predominant hormone receptor status (positive) and human epidermal growth factor receptor 2 (HER2) status (negative) of LS-associated breast cancers were studied for comparison (Table 1). Additionally, existing data from 105 other tumors (including 33 from the colon and rectum, 38 from the endometrium, 13 from the stomach, five from the ureter, five from the bladder, four from the kidney, and seven from the brain) diagnosed in MMR gene mutation carriers were re-evaluated for MMR status and promoter methylation using criteria identical to those presently applied for breast cancer; detailed characteristics of the tumor series can be found in our previous publications [23-25].

**Table 1:** Essential clinicopathological and molecular characteristics of the three breast cancer groups investigated.

	LS-associated breast cancers	LS-associated breast cancers	
	Carriers	Non-carriers	Sporadic breast cancer ^c^
No. of tumors	23	18	49
ductal	17	14	30
ductal *in situ*	2	1	-
lobular	3	-	19
other	1	2	-
no histological data	-	1	-
Size (= 20 mm)	7/19 (37%)	9/14 (64%)	30/49 (61%)
G1	2/19 (11%)	4/12 (33%)	12/46 (26%)
G2	10/19 (53%)	5/12 (42%)	29/46 (63%)
G3	7/19 (37%)	3/12 (25%)	5/46 (11%)
Lymph node metastases	6/16 (38%)	7/10 (70%)	28/47 (60%)
Receptor status:			
ER-positivity	20/22 (91%)	6/7 (86%)	49/49 (100%)
PR-positivity	15/22 (68%)	5/7 (71%)	41/46 (89%)
HER2-positivity	3/20 (15%)	1/6 (17%)	0/49 (0%)
Average age at diagnosis (years)	56	54	61
MSI	8/23 (35%)	0/18 (0%)	N/A
MMR protein reduced or lost	13/20 (65%) ^a^	0/14 (0%) ^b^	0/49 (0%) ^b^
Average number of TSGs methylated out of 24 per tumor	2.3	2.0	2.4

^a^For MMR protein corresponding to the germline mutation; ^b^for any MMR protein investigated (MLH1, MSH2, MSH6); ^c^Clinicopathological data and MMR status reflect selection method used (see text). Note: All frequency calculations are based on tumors for which data were available or which could be successfully analyzed in the laboratory. ER, estrogen receptor; HER2, human epidermal growth factor receptor 2; LS, Lynch Syndrome; MMR, mismatch repair; MSI, microsatellite instability; PR, progesterone receptor; TSG, tumor suppressor gene.

**Table 2:** MMR status and TSG promoter methylation results case by case for breast carcinomas from MMR gene mutation carriers

					MMR protein expression				
Case ID	Predisposing mutation	Histology	Age at diagnosis	MSI status	MLH1	MSH2	MSH6	Overall MMR status^a^	Average number of TSGs methylated out of 24/tumor
2:51	*MLH1 *ex 16: 3,5 Kb deletion (mutation I)	ductal *in situ*	52	MSI	-	+	+	abnormal	1
3:60	*MLH1 *ex 16: 3,5 Kb deletion (mutation I)	ductal	66	MSS	+	+	+	normal	2
10:11	*MLH1 *ex 16: 3,5 Kb deletion (mutation I)	ductal	50	MSS	-	+	ND	abnormal	0
50:44	*MLH1 *ex 16: 3,5 Kb deletion (mutation I)	ductal, left breast	71	MSS	+	+	+	normal	1
		lobular, right breast	72	MSS	-	+	ND	abnormal	4
62:1	*MLH1 *ex 16: 3,5 Kb deletion (mutation I)	ductal + *in situ*	52	MSI	-	ND	ND	abnormal	3
77:15	*MLH1 *ex 16: 3,5 Kb deletion (mutation I)	ductal, right breast	43	MSI	-	+	+	abnormal	0
		lobular, left breast	47	MSS	-	+	ND	abnormal	1
77:24	*MLH1 *ex 16: 3,5 Kb deletion (mutation I)	lobular	51	MSS	+	+	+	normal	0
143:1	*MLH1 *c.454-1G>A, splice site	ductal	56	MSI	-	+	+	abnormal	3
157:1	*MLH1 *c1489insC	ductal	54	MSI	-	+	+	abnormal	4
136:1	*MSH2 *c.1738insA	ductal + *in situ*	79	MSS	ND	ND	ND	ND	2
180:1	*MSH2 *del ex 1-16	ductal	60	MSS	ND	ND	ND	ND	1
191:1	*MSH2 *ex1-7 deletion and *MEN1 *mutation	ductal, right breast, small tumor	48	MSI	+	-	-	abnormal	2
		ductal, right breast, large tumor	48	MSI	+	-	-	abnormal	4
197:1	*MSH2 *c.187delG	ductal	55	MSI	+	-	-	abnormal	1
132:1	*MSH6 *c.2983 G>T, nonsense and *CHEK2 *c.1100delC	ductal *in situ*	69	MSS	+	ND	+	normal	1
132:2	*MSH6 *c.2983 G>T, nonsense and *CHEK2 *c.1100delC	ductal	35	MSS	ND	ND	ND	ND	1
196:1	*MSH6 *ex1-2 deletion	ductal, left breast	52	MSS	+	+	-	abnormal	2
		ductal, right breast	56	MSS	+	+	-	abnormal	3
196:2	*MSH6 *ex1-2 deletion	ductal	56	MSS	+	+	+	normal	2
196:3	*MSH6 *ex1-2 deletion	mucinous	68	MSS	+	+	+	normal	2
196:4b	*MSH6 *ex1-2 deletion	ductal	49	MSS	+	+	+	normal	12

^a^Abnormal MMR status denotes absence of MMR protein corresponding to the germline mutation, presence of MSI, or both. Normal MMR status requires intact MMR protein expression relative to germline mutation and stable microsatellites. NOTE: ND, not defined; for IHC, -, protein absent, +, protein present. IHC, immunohistochemistry; MMR, mismatch repair; MSI, microsatellite instability; TSG, tumor suppressor gene.

DNA was extracted from formalin-fixed paraffin-embedded tumor samples from selected areas with high tumor percentages according to the modified protocol of Isola *et al*. [26]. Normal breast tissue from the same patients was used for the extraction of normal DNA whenever possible. Additionally, to determine the baseline levels of methylation, normal breast tissues from a cohort of unrelated individuals were used as a source of DNA. For protein expression studies, 4 μl sections from breast tumors were cut to glass adhesion slides (Thermo Scientific, Braunschweig, Germany) and air-dried overnight at 37°C. The Institutional Review Boards of Helsinki University Central Hospital (Helsinki, Finland) and Jyväskylä Central Hospital (Jyväskylä, Finland) as well as the National Authority for Medicolegal Affairs (Dnro 1272/04/044/07) approved this study. All necessary consents were obtained from the patients involved.

### MSI analysis

MSI statuses were analyzed using markers from the Bethesda panel [27]. In final interpretations, results from the mononucleotide markers (BAT25 and BAT26) were emphasized in that specimens with one or two unstable mononucleotide repeat markers were considered to have MSI whereas samples with stable mononucleotide repeats were regarded as microsatellite-stable (MSS).

### Immunohistochemical (IHC) staining for MMR protein expression

Breast tumor tissue sections were stained with the following primary mouse antibodies: anti-MLH1 (clone:G168-15, BD Biosciences,/PharMingen, Erembodegem, Belgium) with dilution 1:60, anti-MSH2 (clone:FE11, Calbiochem,/Oncogene Research, Darmstadt, Germany) with dilution 1:80, and anti-MSH6 (clone:44/MSH6, BD Biosciences) with dilution 1:40. The immunohistochemical (IHC) reagents (Dako EnVision+, Dako, Glostrup, Denmark/Carpinteria CA, USA) were utilized according to the manufacturer's specifications. The microwave antigen-retrieval was performed at 750 W for five minutes and 450 W for five minutes in ethylenediaminetetraacetic acid (EDTA) buffer, pH8 (with MLH1 and MLH2 antibodies) and in citrate buffer, pH6 (with MLH6 antibody). The expression results were evaluated as described in Renkonen *et al*. [28].

### Methylation-specific multiplex ligation-dependent probe amplification (MS-MLPA)

The methylation statuses of 24 tumor suppressor genes (TSGs) were analyzed by a commercial MS-MLPA test (SALSA MS-MLPA ME001-C1 Tumor suppressor-1, MRC Holland, Amsterdam,The Netherlands) according to the manufacturer's instructions [29]. The 26 probe pairs included two probe pairs for *MLH1 *of which only the one closest to the transcription start site (1686--L1266) was considered, and two probe pairs for *RASSF1*, where methylation was recorded if one or both showed methylation. DNA (200 ng to 500 ng) from paraffin-derived samples was used. Promega's human genomic female DNA (G152A, Madison WI, USA) was included as a normal reference in every assay. The MS-MLPA test utilizes the methylation sensitive enzyme *Hha1 *(Promega, R6441) which recognizes and digests a CpG site if it is unmethylated [30]. The methylated fraction of the sample will generate an amplified PCR product. The PCR products of individual probes are identified and quantified by capillary gel electrophoresis [31]. The fragment analysis was carried out on the Applied Biosystems ABI 3730 Automatic DNA Sequencer with Applied Biosystems GeneMapper 4.0 genotyping software. The methylation dosage ratios (D_m_) were calculated as described [24]. D_m _≥0.25 (corresponding to at least 25% of methylated DNA) was considered to indicate promoter methylation. This cut-off value provided the best discrimination between tumor DNA and paired normal DNA in the present breast cancer series.

### Statistical analysis

Programs from the VassarStats website [32] were used. The significance level for the differences between groups (*P *value) was determined using Fisher's exact test or Student's t test as appropriate. *P *values <0.05 (two-tailed) were considered significant.

## Results

### Study design and clinicopathological characteristics

This study was based on 90 breast carcinomas (23 from mutation carriers, 18 from non-carriers, and 49 from sporadic cases) (Table 1) and 105 other tumors from MMR gene mutation carriers (see Materials and methods for details). Most breast tumors from mutation carriers were ductal (17 out of 21 invasive tumors, 81%), estrogen receptor (ER) positive (91%), progesterone receptor (PR) positive (68%) and HER2 negative (85%) and the same was true for breast tumors from non-carriers. The hormone receptor and HER2 status of the LS-associated series resembled those in unselected breast carcinomas from our population [33]. The average age at diagnosis was similar in carriers (56 years) and non-carriers (54 years).

### MMR status

**Figure 1:**
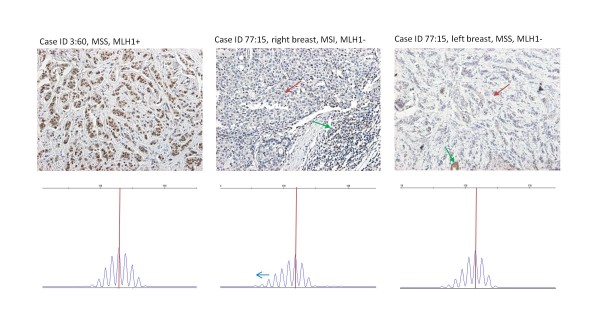
**Outcome of MSI and IHC analyses in three breast tumors from *MLH1 *mutation carriers**. *Left*, MLH1 expressing, MSS breast carcinoma from individual 3:60 (Table 2). *Middle*, ductal carcinoma from the right breast from individual 77:15, displaying MLH1 protein loss and MSI. *Right*, lobular carcinoma from the left breast of the same patient, showing MLH1 protein loss and stable microsatellites. Red arrow in IHC stainings denotes lack of expression in tumor cells and green arrow positive expression in normal cells. MSI results are based on BAT25. IHC, immunohistory; MSI, microsatellite instability; MSS, microsatellite stable.

Detailed molecular data on breast carcinomas from mutation carriers are shown in Table 2. MMR protein corresponding to the germline mutation was reduced or lost in 13/20 (65%) and MSI was present in 8/23 (35%). Examples of positive and negative IHC and MSI results are given in Figure 1. The percentage of MMR-deficient breast carcinomas was lower among *MSH6 *mutation carriers (2/6, 33% by IHC and 0/7 by MSI analysis) compared to carriers of *MSH2 *(3/3, 100% and 3/5, 60%, respectively) or *MLH1 *mutation (8/11, 73% and 5/11, 45%, respectively) (statistically not significant). Breast carcinomas with abnormal MMR by IHC, MSI, or both were diagnosed at an earlier mean age compared to those with normal MMR (53 years versus 61 years, *P *= 0.027). Abnormal MMR was significantly more frequent in breast carcinomas from mutation carriers than in those from non-carriers (13/20, 65% versus 0/14, 0%, *P *= 0.00016 by Fisher's test) (Table 1).

### TSG promoter methylation

**Figure 2:**
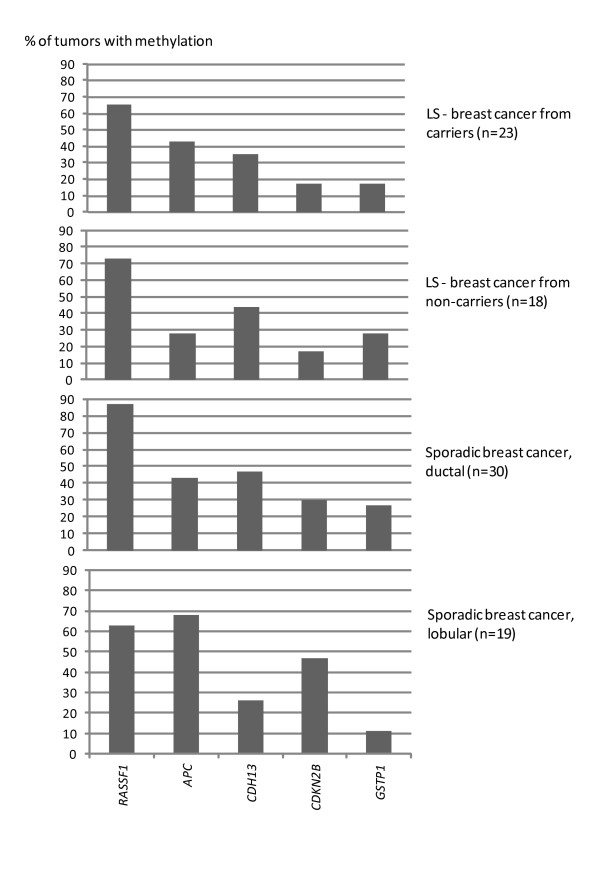
**TSG methylation profiles in breast tumors from MMR gene mutation carriers, non-carriers and sporadic cases**. Breast carcinomas from MMR gene mutation carriers and non-carriers were mostly ductal (Table 1) whereas sporadic cases were divided into ductal and lobular subgroups. Only TSGs which showed promoter methylation in at least 10% of tumors from any group (carriers, non-carriers, sporadic) were included in this comparison. MMR, mismatch repair; TSG, tumor suppressor gene.

Among 24 TSGs investigated, the average number of genes showing promoter methylation in breast carcinomas from mutation carriers was 2.3 per tumor, which was comparable to non-carriers and sporadic cases (Table 1). The most frequently methylated genes were *RASSF1 *(65%), *APC *(43%), *CDH13 *(35%), *GSTP1 *(17%), and *CDKN2B *(17%), and the pattern was quite similar in breast carcinomas from mutation carriers, non-carriers and sporadic ductal cases (Figure 2). Ductal and lobular breast cancers showed some differences in relative frequencies of methylation at promoters of individual TSGs (Figure 2). In particular, *CDKN2B *methylation was more common in lobular than ductal breast carcinoma at a borderline level of significance (10/22, 45% versus 14/64, 22% with all groups included, *P *= 0.052 by Fisher's test).

### Comparison of different tumors from MMR gene mutation carriers

**Figures 3:**
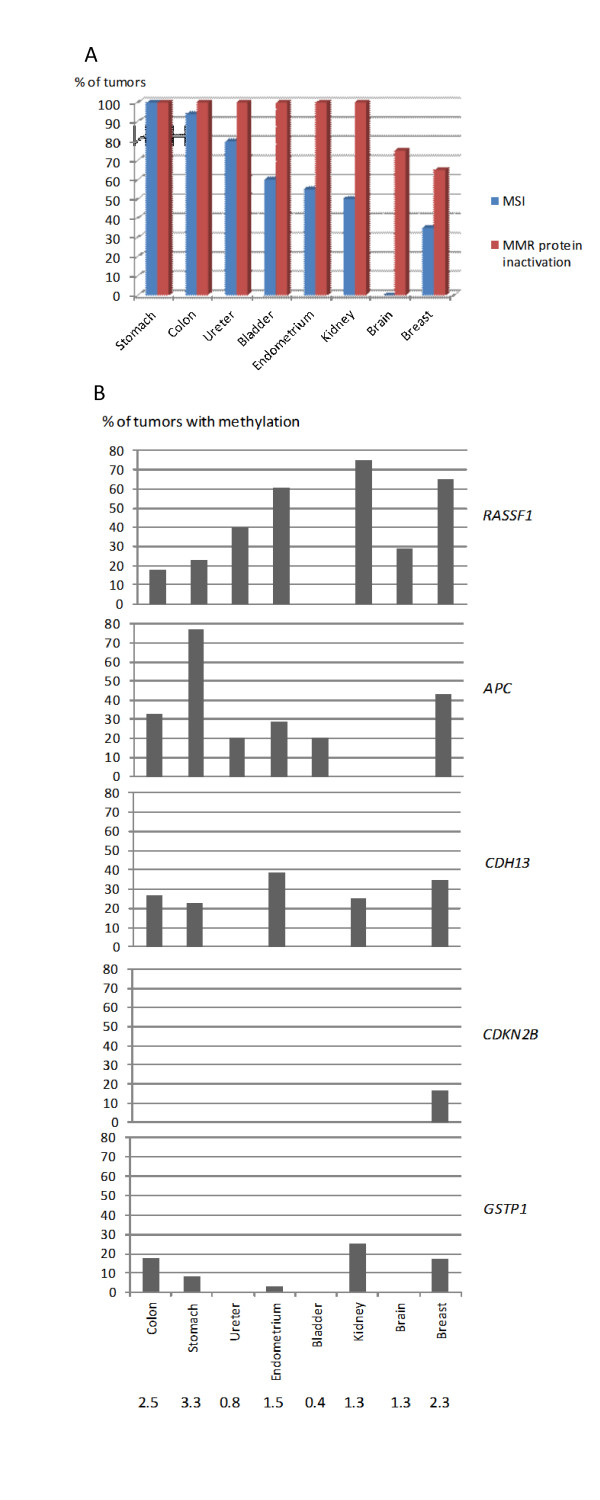
**MMR status and TSG methylation in eight different tumor types from mutation carriers**. **A**, for MMR status comparisons, the frequencies of MSI and abnormal IHC in the same tumors are indicated. **B**, Among TSGs, the top five loci affected by methylation in breast cancer were chosen for display. Dm >0.25 was used as a cut-off for methylation for all tumors. The average number of methylated TSGs out of 24 per tumor is given below each tumor type. Dm, methylation dosage ratio; IHC, immunohistochemistry; MMR, mismatch repair; MSI, microsatellite instability; TSG, tumor suppressor gene.

Evaluation of the MMR status in eight different tumor types from MMR gene mutation carriers showed that breast carcinoma had the lowest frequency of MMR protein inactivation and the second lowest frequency of MSI (Figure 3A). TSG promoter methylation revealed organ-specific profiles (Figure 3B). The top five genes affected by methylation in breast carcinoma were involved with variable frequencies in the other tumor types, too, except for *CDKN2B *which was methylated in breast carcinoma only. In regard to the average number of methylated TSGs out of 24 per tumor, breast carcinoma (2.3) was the third from top (after 3.3 for stomach and 2.5 for colon cancer; Figure 3B).

## Discussion

Genetic and clinical heterogeneity characterize LS and manifest themselves in the wide spectrum of cancers that can be found in LS families [1,34]. We set out to address whether breast carcinoma is part of the LS tumor spectrum based on molecular characteristics of the individuals and tumors. To our knowledge, our study is the first to compare breast carcinomas from mutation carriers to those from proven non-carriers (phenocopies) and to sporadic breast carcinomas from the same population. This setting provides a reliable reference for the background rates of the alterations examined. For example, many cancers that occur in LS are also common in the general population, reflecting the combined effect of low-penetrance susceptibility alleles and environmental factors [35]. Moreover, the pattern of organ involvement has changed over time including, for example, a relative decline in stomach cancer and an increase in colorectal cancer incidence in more recent generations [36] and may vary according to the ethnic or geographic origin [13,37], suggesting that the LS tumor spectrum is sensitive to environmental influences. The present utilization of representative sets of other tumors from carriers of the same mutations additionally makes it possible to recognize the important role that the tissue of origin may play as a mediator of the effects of MMR deficiency [38] or epigenetic dysregulation [39]. Finally, previous molecular studies on LS-associated breast carcinoma have mainly focused on the MMR status; we additionally evaluated the epigenetic profiles of the tumors.

Our previous epidemiological investigation of MMR gene mutation carriers from Finnish LS families [5] arrived at a standardized incidence ratio of 1.4 for breast cancer compared to the general population, suggesting that breast cancer incidence is not elevated in Finnish LS families. This finding is in agreement with most reports from other populations (see Introduction). In the present study, breast carcinoma was diagnosed at 56 years of age in carriers and 54 years in non-carriers, which is comparable to sporadic breast carcinomas from our population (59 years) [33]. In the available literature, the average age of breast carcinoma diagnosis in MMR gene mutation carriers varies between 46 years and 66 years, including both early-onset series [6,18,40] and series with the age at onset similar to the general population [8,17]. Interestingly, the age at diagnosis was significantly lower for MMR-deficient- (53 years) versus MMR-proficient breast carcinomas (61 years) among mutation carriers from our study. Combined with the normal life-time risk of breast cancer in our LS families, the finding is compatible with the idea that MMR defects might preferentially be involved in breast cancer progression rather than initiation [6]. The breast carcinomas we studied from MMR gene mutation carriers were predominantly ductal, ER- and PR-positive and HER2-negative, resembling breast carcinomas from non-carriers (Table 1) and those from the unselected Finnish population [33,41]. Similar to our findings, ductal histology predominates in published reports on LS-associated breast carcinomas [17-19]. While the hormone receptor or HER2 status has seldom been specified in previous papers, a single study [19] found predominant hormone receptor negativity among MMR-deficient breast cancers from MMR gene mutation carriers; furthermore, there was no difference in the age at onset between MMR-deficient and MMR-proficient breast cancers. Apart from possible population-specific differences, reasons for the conflicting results relative to ours are unknown.

Apparent selectivity in tumor spectrum despite ubiquitous expression is a puzzle shared by a majority of high-penetrance susceptibility genes, including the MMR genes in LS. In theory, the LS tumor spectrum could be influenced by the predisposing gene and mutation (for example, carriers of *MSH2 *mutation may have a higher risk of various extracolonic tumors compared to *MLH1 *mutation carriers [6]). MMR gene dosage, which affects the extent and degree of the MMR defect, is also important, both constitutionally (for example, biallelic germline mutations are associated with a distinct tumor spectrum characterized by hematological and central nervous system malignancies, [42]) and somatically (for example, DNA damage signaling requires a higher dosage of MMR protein than DNA mismatch repair [43] and inactivation of both alleles of a MMR gene may, therefore, not always be necessary in tumor cells). Different target genes are rate-limiting in different cancers and their susceptibility to mutations and selective advantage conferred by the mutations may vary (for example, *TGFBRII *and *PTEN *both contain coding repeats which are structurally prone to frameshift mutations in the context of deficient MMR, but *TGFBRII *is mainly involved in gastrointestinal cancers and *PTEN *in endometrial cancer [44]. Finally, different organs may be differently exposed to exogenous (for example, dietary carcinogens) or endogenous agents (for example, hormone-induced oxidative stress) and removal of such damage may depend on functional MMR [45,46].

In our study, 65% (13/20) of breast carcinomas from MMR gene mutation carriers showed loss of the MMR protein corresponding to the germline mutation and 35% (8/23) had high-degree MSI. In comparison with other tumors from MMR gene mutation carriers, the percentage of breast carcinomas with MMR protein inactivation was consistently lower and the proportion with MSI-H in the lower range (Figure 3A). The fact that *MSH6 *mutations were overrepresented among patients with breast carcinomas (7/23, 30%) versus other tumors (4/105, 4%) might have some influence on the results since the percentage of MMR-deficient breast carcinomas was lower among *MSH6 *than *MSH2 *or *MLH1 *mutation carriers (Table 2). Importantly, we observed that a given MMR gene mutation could be associated with MMR proficient breast carcinomas in some carriers and MMR-deficient breast carcinomas in other carriers of the same mutation (see Table 2 and Figure 1 for examples), ruling out the possibility that normal protein expression or microsatellite-stability was a general characteristic of these mutations. We conclude that breast carcinomas from MMR gene mutation carriers conformed to other LS tumors in that IHC analysis of MMR protein expression was more sensitive than MSI analysis to detect a MMR defect but deviated from the remaining tumors in that even IHC failed to detect a MMR defect in a considerable proportion (35%) arguing against biallelic inactivation in those cases. Even so, the proportion of MMR-deficient breast carcinomas was significantly higher compared to breast carcinomas from non-carriers (Table 1) suggesting that MMR defects do play a role in breast tumorigenesis in MMR gene mutation carriers. Furthermore, as discussed above, the possible role of MMR gene malfunction in processes other than MMR cannot be completely excluded in the case of MMR-proficient tumors.

The role of growth-regulatory target genes was addressed through studies of promoter methylation, which is known to be an important mechanism of inactivation of the TSGs examined [25,47]. The average number of methylated TSGs out of 24 was in the upper range in breast carcinomas among all tumors from MMR gene mutation carriers (Figure 3B). The five most frequently methylated TSGs (*RASSF1, APC, CDH13, GSTP1*, and *CDKN2B*) were the same in all groups of breast cancer (Figure 2) and, with the exception of *CDKN2B*, were involved in other tumors from LS patients, too, although with variable frequencies (Figure 3B). *CDKN2B *promoter methylation may be a particular characteristic of breast tumorigenesis as also supported by observations from other groups [48].

## Conclusions

Our findings combined with available literature suggest that breast carcinoma arising in MMR gene mutation carriers resembles common breast carcinoma with respect to life-time risk, clinicopathological features, and TSG methylation profiles. Significantly higher frequencies of MMR-deficiency distinguish breast carcinomas from MMR gene mutation carriers from those from proven non-carriers and sporadic cases. The data as a whole suggest an association between breast cancer and inherited MMR deficiency (LS), but appropriate caution is warranted in interpretations because of the relatively small numbers of cases investigated and the variability between individual studies. Clinically, the results are important for the establishment of LS diagnosis especially in families lacking typical LS spectrum tumors; in such cases, IHC analysis of breast carcinoma may be valuable to pinpoint the predisposing gene. The fact that breast carcinoma may develop as a consequence of the predisposing MMR gene defect needs to be taken into account in the counseling of female MMR gene mutation carriers [49]. Mutation carriers should be encouraged to actively participate in population-based breast screening programs, which typically target women aged 50 years and over [50].

## Abbreviations

*APC*: human adenomatous polyposis coli; *CDH13*: human cadherin 12/H-cadherin; *CDKN2B*: human cyclin-dependent kinase inhibitor 2B; Dm: methylation dosage ratio; ER: estrogen receptor; *GSTP1*: human glutathione S-transferase pi 1; HER2: human epidermal growth factor receptor 2; IHC: immunohistochemistry; MSS: microsatellite stable; LS: Lynch syndrome; MMR: mismatch repair; *MLH1*: human mutL homolog 1; *MSH2*: human mutS homolog 2; *MSH6*: human mutS homolog 6; MSI: microsatellite instability; MS-MLPA: methylation-specific multiplex ligation-dependent probe amplification; PCR: polymerase chain reaction; *PMS2*: human post meiotic segregation increased homolog 2; PR: progesterone receptor; *PTEN*: human phosphatase and tensin homolog; *RASSF1*: human ras association (RalGDS/AF-6) domain family member -1; *TGFBRII*: human transforming growth factor: beta receptor II; TSG: tumor suppressor gene.

## Competing interests

The authors declare that they have no competing interests.

## Authors' contributions

JEL performed all methylation studies for breast tumors. AG conducted IHC analyses for breast tumors. AG, TTN, and JEL were responsible for MSI analyses. TK, WA-R, RP and MF contributed to histological analyses of tissue specimens and evaluated IHC results. J-PM, HJ, MA and KA diagnosed and recruited the Lynch syndrome cases and TK provided specimens from the sporadic cases. PP, J-PM, TK, and JEL conceived the study and drafted the manuscript. All authors read and approved the final manuscript.
